# Langerhans cell sarcoma with an aberrant cytoplasmic CD3 expression

**DOI:** 10.1186/1746-1596-7-128

**Published:** 2012-09-25

**Authors:** Zhaodong Xu, Ruth Padmore, Carolyn Faught, Lisa Duffet, Bruce F Burns

**Affiliations:** 1 Department of Pathology and Laboratory Medicine, Division of Hematopathology, The Ottawa Hospital, 501 Smyth Road, Ottawa, Ontario, Canada; 2 Department of Medicine, Division of Hematology, The Ottawa Hospital, 501 Smyth Road, Ottawa, Ontario, Canada; 3 Department of Pathology and Laboratory Medicine, Division of Anatomical Pathology, The Ottawa Hospital, 501 Smyth Road, Ottawa, Ontario, Canada

**Keywords:** Langerhans cell sarcoma (LCS), Langerhans cell histiocytosis (LCH), CD3, Aberrant expression, Lineage plasticity, Transdifferentiation

## Abstract

**Abstract:**

Langerhans cell sarcoma is a rare and aggressive high grade hematopoietic neoplasm with a dismal prognosis. It has a unique morphological and immunotypic profile with a CD1a/ langerin/S100 + phenotype. T cell lineage markers except for CD4 in Langerhans cell sarcoma have not been documented previously. We report a case of 86 year-old male of Caucasian descent who presented with an enlarging right neck mass over 2 months with an underlying unknown cause of anemia. Computed tomography scan of the neck, chest and abdomen revealed generalized lymphadenopathy and mild splenomegaly suspicious for lymphoma. Diagnostic core biopsy performed on right neck mass revealed a possible T cell lymphoma with expression of T cell lineage specific marker CD3 but conclusive diagnosis could not be made due to insufficient core biopsy sample. Further excisional biopsy performed on a left inguinal node showed a hematopoietic neoplasm with features of Langerhans cell sarcoma with a focal cytoplasmic CD3 expression in 30-40% of the tumor cells. PCR for T cell receptor (TCR) gene rearrangement failed to demonstrate a clonal gene rearrangement in the tumor cells arguing against a T cell lineage transdifferentiation, suggesting an aberrant CD3 expression. To the best of our knowledge, this case represents the first report of Langerhans cell sarcoma with an aberrant cytoplasmic CD3 expression.

**Virtual slides:**

http://www.diagnosticpathology.diagnomx.eu/vs/2065486371761991

## Background

According to the most recent WHO Classification of Tumors of Hematopoietic and Lymphoid Tissues (2008) [1], Langerhans cell sarcoma (LCS) belongs to the category of histiocytic and dendritic cell neoplasms. It is a rare subtype of tumors derived from Langerhans cells with a female predominance.

Clinically, LCS is often an extranodal tumor with skin and bone involvement, but it may present with multi-organ involvement including lymph node, lung, liver and spleen. 11% of the reported cases patients had pancytopenia. Compared to more commonly known Langerhans cell histiocytosis (LCH), also a clonal neoplastic proliferation of Langerhans cells, LCS has a much higher degree of cytological atypia and prominent proliferation rate with an aggressive clinical course. Both LCH and LCS have a similar immunophenotype including CD1a/langerin/S100+ [2,3] but without B- and T-cell lineage markers except for CD4 [4]. LCS with a cytoplasmic T cell specific marker CD3 has heretofore not been reported in the English literature. Herein we provide the first report of LCS with an aberrant cytoplasmic CD3 expression.

## Case presentation

An 86 year old Caucasian male who had been relatively healthy with medically controlled diabetes on insulin, treated temporal arteritis and myocardial infarction in 1987 without ongoing angina was referred to hematology/Oncology service for possible T cell lymphoma at Ottawa Hospital in July 2011. The patient had a short history of a rapidly growing right neck mass, an anemia of unknown cause, mild weight loss and generalized weakness. Laboratory investigations showed a bicytopenia with a normocytic anemia, Hb 89 g/L (reference interval 115–155 g/L) and thrombocytopenia, PLT 86 x 10^9^/L (reference interval 125–400 x 10^9^/L). He also had a neutrophilia with WBC 19.8 x 10^9^/L (reference interval 3.0-10.5 x 10^9^/L). LDH was mildly elevated with a value of 240 u/L(reference interval 100–205 u/L). CT of neck/thorax/abdomen demonstrated diffuse lymphadenopathy involving the neck, mediastinum, lung, abdomen and pelvic and mild splenomegaly. Two previous right neck core biopsies at another hospital suggested a peripheral T cell lymphoma based on the expression of T cell specific marker CD3 with negative CD20 and cytokeratin AE1/3. Definitive diagnosis could not be made due to insufficient tissue. An excisional left inguinal node biopsy was performed at the Ottawa Hospital.

**Figure 1:**
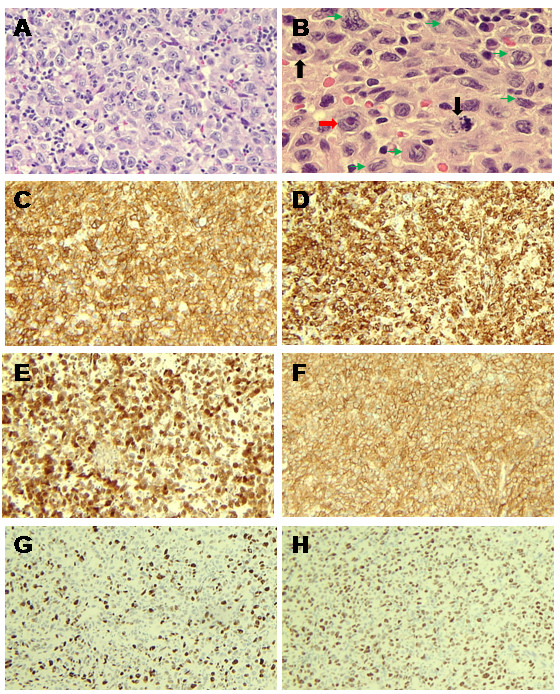
**H & E staining.A**. Intermediate power view showing lymph node was replaced by a population of large cells with scattered background small lymphocytes. **B**. High power views of tumor cells demonstrated typical nuclear groove (green arrows), prominent nucleoli (red arrow), and abundant eosinophilic cytoplasm. Mitotic figures can be easily found (black arrows). Immunohistochemistry. **C**-**H**. Tumor cells demonstrated strong positivity for CD1a (**C**), langerin (**D**), S100 (**E**) and CD4 (**F**). Tumor cells showed positivity for cell cycle regulator p53 (**G**). Tumor cells were labeled more than 50% of cell proliferation index marker Ki-67 (**H**).

Microscopic evaluation of formalin-fixed, paraffin-embedded lymph node tissue revealed diffuse architectural effacement of the lymph node with replacement by a population of large hematolymphoid cells with very distinctive nuclear features. The nuclei were slightly elongated and many of them showed prominent longitudinal nuclear grooves reminiscent of Langerhans cells (Figure 1 A). These cells however were larger, had more prominent nucleoli, abundant eosinophilic cytoplasm and a higher degree of pleomorphism than normal Langerhans cells and occasional cells were multinucleated. The cells were growing in sheets and there were very few admixed lymphocytes or eosinophils in the background. Scattered mitotic figures were easily appreciated. (Figure 1 B).

The immunohistochemical profile of the case is summarized in Table 1. The tumor cells showed intense membrane staining for CD1a, strong granular cytoplasmic staining for langerin (CD207), strong staining for S100 and CD4. (Figure 1 C, D, E, F). The Ki-67 proliferation index in tumors cells was >50% (Figure 1G). p53, a cell cycle regulator (Figure 1H) and bcl-2 were also positive. CD30 was focally positive in about 30-40% of the tumor cells (see Table 1).

**Table 1:** Immunohistochemical staining of Langerhans cell sarcoma

**Marker**	**Langerhans cell sarcoma in our case**	**Typical immunophenotype of tumors derived from Langerhans cells**
**CD1a**	+	+
**Langerin**	+	+
**S100**	+	+
**CD4**	+	+
**CD3**	30-40% + cytoplasm	-
**p53**	+	+/−
**CD30**	30-40% +	NK
**BCL2**	+	+
**Ki-67**	>50%	0-25%
**CD5**	-	-
**CD8**	-	-
**CD10**	-	-
**CD20**	-	-
**CD23**	-	-
**CD56**	-	-
**BCL6**	-	-
**MUM-1**	-	-
**Cyclin D1**	-	-
**CD68**	-	+/−
**CD163**	-	NK
**ALK**	-	-

NK: not known.

**Figure 2:**
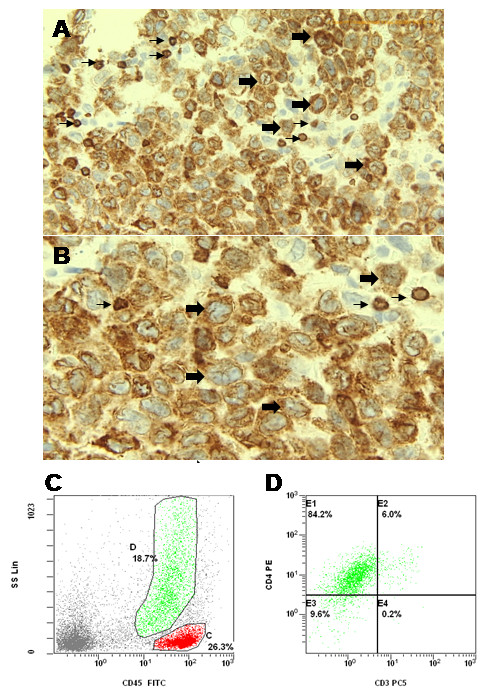
**T cell specific marker CD3 staining and Lymph node flow cytometry.A**. Low power view and **B**. high power view showing tumor cells had focal cytoplasmic CD3 staining (large arrows) in contrast to background small T lymphocytes with strong membrane CD3 staining (small arrows). **C**, **D**. Lymph node flow cytometry immunophenotyping showed 2 populations of cells, with normal lymphocytes (in red) and an abnormal population with high side scatter (in green) which show expression of CD4 and are negative for surface CD3. Cytoplasm CD3 was not done.

Interestingly, specific monoclonal antibody CD3 against epsilon chain of human CD3 complex (LN10, Catalog No: PA0553, Leica Biosystems) showed a focal cytoplasmic staining in 30-40% of the tumor cells, comparing to the strong staining of admixed normal T lymphocytes (Figure 2 A, B). The morphology and immunohistochemical profile of this tumor strongly suggested a Langerhans cell derived neoplasm. Although double staining immunohistochemistry was not performed, with the extensive degree of staining with CD1a, S100, langerin, CD3 and CD30, the tumor cells were interpreted as positive for all these markers (see Figure 1 and Table 1). The high mitotic rate and high labelling with Ki-67, along with p53 expression, coupled with the significant cytological atypia, rare background eosinophils indicated a diagnosis of Langerhans cell sarcoma. Flow cytometry profiling of the lymph node showed 2 population of cells, a small cell population in the lymphocyte gate showing no abnormal expression of aberrant B and T cell markers, and an abnormal cell population evidenced by a high side scatter in flow histogram, which demonstrated CD4 expression (Figure 2 C, D). This was compatible with immunohistochemical findings of CD4 positivity of tumor cells and scatter normal lymphocytes at the background in the tissue block. Cytoplasmic CD3 was not done on flow cytometry but the abnormal cells were negative for surface membrane CD3. Bone marrow aspiration and biopsy did not show bone marrow involvement. Therapy for LCS may be toxic and anti-CD30 therapy (SGN-30) may have modest clinical activity [5,6]; based on the aggressiveness of the disease, patient’s age, and poor ECOG performance status [7] with a score 3–4 out of 5, palliative measures with local radiotherapy were instituted after discussion with the patient and his family. Patient died one month later after pathological diagnosis.

## Discussion

Langerhans cells are one subset of hematopoietic cells, thought to derive from monocyte-macrophage lineage. They are specialized dendritic cells in skin or mucosal sites devoted to antigen presentation to T cells upon activation. They are then thought to migrate to lymph node through lymphatics. Physiologically, Langerhans cells can respond in a non-clonal fashion to certain reactive stimuli such as smoking, in the lung [8,9]. Alternatively, they can proliferate in a clonal pattern, forming tumors designated as LCH and much less commonly as LCS. LCH can also be associated with other disease processes such as malignant lymphoma, and myasthenia gravis [10,11], but there is still debate about whether these represent a clonal or non-clonal reactive process in those situations.

LCH and LCS have been believed to originate from myeloid stem cells [12-16] rather than lymphoid stem cells, but some recent experimental and clinical evidence has argued against this belief [17,18]. Furthermore, some clinical reports have suggested that both B- and T-cell neoplasms can transdifferentiate into LCH and LCS after prolonged treatment of the original disorders [19-23], suggesting that in these hematolymphoid neoplasms the tumor cells have some potential for lineage plasticity, somewhat comparable to the leukemias with lineage plasticity, acute leukemias of ambiguous lineage in the 2008 WHO hematolymphoid tumour classication. Because of its linkage to the T-cell receptor molecules surface membrane CD3 is felt to be the most specific T lineage marker along with cytoplasmic epsilon chain, although CD3 epsilon chain can be expressed in cytoplasm of NK cells and thymocytes. Furthermore aberrant CD3 expression has been reported in other hemotopoietic tumours such as diffuse large B cell lymphoma, primary mediastinal large B cell lymphoma, plasmablastic lymphoma and classical Hodgkin’s lymphoma [24-27] but not in histiocytic sarcoma. In addition, LCH can co-exist with T cell lymphoblastic lymphoma in the same tissue, making the correct diagnosis difficult [28,29].

In our case, because of the expression of cytoplasmic CD3 and CD4, we performed T cell receptor (TCR) gene arrangement studies for gamma, delta and beta chains by PCR to rule out the possibility of T cell lineage transdifferentiation or lineage plasticity. The results failed to demonstrate a clonal rearrangement pattern, suggesting that the LCS tumor cells had an aberrant cytoplasmic CD3 expression rather than a T cell transdifferentiation phenomenon.

As a rare subtype of sarcoma, LCS has a poor prognosis [30]. Although the prognostic factors in LCS were not known, they might include prognostic factors common to other rare sarcomas, such as patient age, tumor size, tumor cell grade, proliferation rate, and/or stage. [31-33]. DNA ploidy had been proposed as one of the prognostic factors in some subtypes of sarcoma [31,34], although another study does not show ploidy status to be an independent prognostic factor [35]. The prognostic role of DNA ploidy in LCS had never been explored. In our case, due to the rarity of LCS and finesse of the technique, DNA ploidy study was not performed.

Among the reported LCS cases [1], patients had a predominantly female distribution with a median age of 39 years old, ages ranging from 10 to 72 years. Our patient, at 86 was thus the oldest reported to date.

## Conclusion

In summary, we reported the first case of Langerhans cell sarcoma with an aberrant cytoplasmic CD3 expression, which initially with insufficient immunophenotyping created diagnostic difficulty, especially about lineage assignment. Recognizing the cytomorphologic features characteristic of Langerhans cells prompted use of markers enabling the correct diagnosis.

## Consent

Written informed consent was obtained from the patient for publication of this Case Report and any accompanying images. A copy of the written consent is available for review by the Editor-in-Chief of this journal.

## Competing interests

The authors declare that they have no competing interests.

## Authors’ contributions

The manuscript was prepared by ZX under the supervision of BFB. BFB and ZX were directly involved in diagnosis and interpretation of histology and immunohistochemistry results. RP were responsible for interpretation of flow cytometry results and paper discussion. CF and LD were responsible for the treatment of the patient. All authors read and approved the final manuscript.
